# An efficient data sheet based parameter estimation technique of solar PV

**DOI:** 10.1038/s41598-024-57241-5

**Published:** 2024-03-18

**Authors:** K. M. Charu, Padmanabh Thakur, Nikita Rawat, Fahim Ansari, Sandeep Gupta, Mukesh Kumar

**Affiliations:** 1 Department of Electrical Engineering, Graphic Era (Deemed to be University), Dehradun, 248002 Uttarakhand India; 2 Department of Mechanical Engineering, Assosa University, Assosa, Ethiopia; 3 Dev Bhoomi Uttarakhand University, Dehradun, India

**Keywords:** Renewable energy, Engineering, Electrical and electronic engineering

## Abstract

This work develops an efficient parameter estimation technique, based on manufacturer datasheet, to obtain unknown parameter of solar photovoltaic (PV), precisely. Firstly, a nonlinear least square objective function, in terms of variables given in manufacturer datasheet, has been developed. Then, two optimization techniques, namely the Particle Swarn Optimization (PSO) and Harmony Search (HS) are applied on the developed objective function to achieve the optimized result. Further, the correctness of the developed technique is tested by estimating the performance indices, namely percentage maximum power deviation index (%MPDI) and overall model deviation index (OMDI), of two different solar PV, viz., Kyocera KD210GH-2PU (poly-crystalline), and Shell SQ85 (mono-crystalline). It is shown that developed method with PSO outperforms the HS. The developed method with PSO gives the values of %MPDI and OMDI of 0.0214% and 0.213, only. Also, the existing methods, based on hybrid, multi-objective function, numerical method, have been considered for the comparative analysis. It is revealed through the comparative studies that the developed method with PSO has smaller value of MPDI (= 0.0041%) and OMDI (0.005) than the other existing methods. Further, the convergence of the developed method has also been estimated to check the speed of estimation. It is shown that the developed technique converges only in 5 s. In addition, the developed technique avoids the need of extensive data as it is based on manufacturer datasheet.

## Introduction

Nowadays, the solar PV systems are being recognized as the immerging and promising potential source of electrical power generation due to their characteristics, namely nondepletable, indigenous, flexible size, and virtually non-polluting. Furthermore, due to the limited spaces requirement for their installation, such as the rooftops and side of the buildings, making it more reachable to both commercial as well as domestic users. Unfortunately, due to significant installation cost and suboptimal conversion efficiency, it faces the challenges in terms of competitiveness against the conventional electrical generation systems^1^.

Therefore, in order to get efficient design of solar PV systems, its characterization through simulation and emulation became crucial before proceeding to the installation stage^2^. An accurate emulation of the solar PV cell, done beforehand the installation and operation, can aid in designing a high-performance controller^2^. Additionally, it can aid in optimizing the PV system operations by anticipating the exact output power that is yielded by the solar PV plant during different environmental conditions. The characteristics of solar cell at varying environmental condition is desirable for the emulation of solar PV. Further, in varying environmental condition, the estimation of unknown parameters becomes a challenging task due to shift in the characteristics with the environmental conditions^2,3^.

Several mathematical structure of solar PV, including single exponential model (SEM), double exponential model (DEM), fuzzy logic models, and ANN based model have already been studied in the research for the exact analysis of solar PV^1,4^. However, each of these models has their own merits and limitations. However, due to the simplicity of SEM, it has greatly been recognized for the characterization^5–10^. Moreover, considering the simplicity of SEM, the myriads of researches, based on analytical, numerical, metaheuristic, and hybrid approach, were also carried out to achieve its accurate characteristics^11–31^. Though, these methods are able to get acculturate characteristics but on the other hands faces challenges of the requirement of extensive experimental data for the validation of effectiveness and accuracy. Furthermore, low convergence rate, proper selection of initial value, large numbers of unknown variables, and use of assumptions & approximations are the other few major limitation of these methods^1,5,8^. Usually, the knowledge of slope, i.e*.,* at the short circuit region $\left( {{\text{R}}_{{{\text{sho}}}} = - \left. {\frac{{{\text{dV}}}}{{{\text{dI}}}}} \right|_{{{\text{I}} = {\text{I}}_{{{\text{sc}}}} }} } \right)$ and at the open circuit region $\left( {R_{SO} = \left. { - \frac{dV}{{dI}}} \right|_{{V = V_{OC} }} } \right)$, are very crucial for the analytical and numerical methods^8–10^. Here, $R_{sho}$ is the shunt resistance at short circuit region and $R_{so}$ is the series resistance at open circuit voltage. Moreover, the use of assumptions and approximation, in the analytical methods, makes characteristics very far from the real. For instant, in most of the work, based on analytical methods, the value of ‘*A*’ is taken in the range of 1–1.5, the value of ‘*R*_*sh*_’ is considered as infinity, the value of ‘*R*_*s*_’ is considered as zero, and *I*_*pv*_ is taken as *I*_*sc*_^1,8^. Though, these assumptions reduce the complexity but on the other side improves the inaccuracy in real characterization^1^. Indeed, these unknown model parameters are existing due to the inherent property of the cell materials and hence their accurate estimation play very crucial role to get enhanced efficiency. In nutshell, it can be said that, the inaccurate estimation of these unknown model parameters may lead to the erroneous characterization.

Usually, numerical methods use the iterative techniques. The iterative techniques, such as Newton Raphson^11^ and Gauss Sidal^12^, require extensive experimental data for the optimization of the error between estimated and experimental *I–V* curve^13^. Also, a similar approach has been used by metaheuristic optimization algorithms-based methods and hence faces same challenges as in the numerical methods. Additionally, for PV modules consisting of numbers of PV cells, it becomes hectic to accumulate experimental data, especially for small solar PV systems for domestic purposes. In addition to the limitations, as mentioned in aforementioned discussions, the parameter estimation methods, based on experimental data, are only suitable for the particular solar PV cell for which data are accumulated. Thus, there is a need to develop a general parameter estimation method that can be used on any PV module by just utilizing the manufacturer’s information.

Moreover, the hybrid parameter estimation methods consider the hybridization of either ‘numerical & analytical’ or ‘numerical & iterative’ or ‘numerical & metaheuristics’-based technique^1,8,14–16,27–29^. For instance, Newton Raphson and Levenberg–Marquardt damping parameter are combined in^27^ to get accurate values of unknown parameters of SDM and DDM model of solar PV. In the same line, hybridization of opposition-based learning reptile search algorithm and Cauchy mutation strategy is presented in^28^, a hybrid approach, based on modified third order Newton Raphson has been given in^29^. Recently, some recent artificial intelligent techniques, such as sanitized teacher learning-based optimization^30^, jellyfish search optimizer^31^, success-history adaptation differential evolution with linear population size reduction^32^ have also been in the research to get accurate values of the unknown parameters.

As discussed in aforementioned studies, the limitations of the ‘Analytical’, ‘Iterative’, and ‘Hybrid Approach’ can be summarized in the following points^27–32^:

1. The accuracy of the analytical method is greatly dependent on the accuracy of the solution of the non-linear equations which are obtained at the remarkable points. As the equations are nonlinear and so any error in the solution may results in erroneous characterization.
2. Usually, assumptions and approximations were considered in the analytical methods to make calculation simple. Therefore, the characteristics, as obtained with the analytical methods, differ from the real characteristics.
3. However, iterative techniques can be used to overcome the limitations existing in the analytical methods. But on the other hand, the selections of the initial guess are the challenging task for these methods. Further, third order Newton Raphson is found to be suitable only for triple diode model of solar PV. Additionally, disturbance in the solution and slow convergence are seen in higher order Newton Raphson method.
4. However, the hybrid approach provides better characterization than ‘numerical, iterative’ and ‘metaheuristics’-based technique but on the other side hybridization of two techniques enhances the computational burden.
5. Furthermore, in the hybrid approach, the anomalies, such as slow convergence, selection of the initial guess, the limitations existing due to model non-linearity, remove partly but not the completely. In addition, the most of the hybrid approaches are still based on assumptions and approximations.

Additionally, the most of the methods, as discussed in aforementioned studies, are developed and validated using the experimental data. As discussed, the experimental dataset-based methods are only suitable for the specific solar PV and these methods cannot be considered as the general method. Moreover, the merits of datasheet-based approach of the parameter estimations over experimental based approach can be summarized with the following points:

1. The data, which are required to get unknown parameters, are readily available and reliable as it is estimated under controlled conditions.
2. Experimental based parameter estimation methods require extensive experiment and hence time consuming and costly. Further, the environmental conditions are not constant throughout the day and hence not reliable.
3. The data provided by the manufacturer along with temperature and insolation intensity, it is possible to predict the ability of electricity generation under different environment conditions.
4. The datasheet-based parameter technique estimates the unknow parameters more quickly than the experimental based method due to availability of data in standard format. Further, no any data collections are required like in experimental based methods.
5. Experimental based parameter estimation techniques are specific while the datasheet-based methods are general and can be used for any PV modules.

Therefore, taking into account, its simplicity, speed of estimation, cost, reliability, and stability, this work develops a novel technique for the determination of the unknown parameters of solar PV using manufacturer datasheet. In the developed work, firstly datasheet based non-linear least square (NLS) objective function has been developed and then PSO and HS algorithms are used for its optimization. The developed datasheet method has the following attributes:

1. Collections of extensive *I-V* data do not require like in experimental based parameter estimation methods.
2. The developed method requires data on three remarkable points, i.e*., V*_*oc*_, *I*_*sc*_*,* and *MPP*. These data are given in datasheet. Therefore, the developed method is reliable and fast.
3. The developed method does not consider any assumption and approximation and hence the characteristics closely align with real characteristics of solar PV.
4. Absolute relative maximum power error ($E_{ARMP} {\text{\% }})$ and overall model error ($E_{OME}$) are found smaller than the experimental based method.
5. Finally, the developed method alleviates the complexity of parameter estimations and hence mitigates the need for assumption and approximation.

This paper is structured as follows: the discussion about solar PV model has been presented in Section “Solar PV model” whereas section “Development of the objective function based on datasheet” covers the development of the NLS objective function based on datasheet. Additionally, a brief discussion about the metaheuristic methods, namely PSO and HS have also been incorporated in section “Development of the Objective function based on datasheet”. The findings of the developed method and comparative analysis with some other established methods were incorporated in section “Results and discussions”. Finally, conclusions of the developed work are given in section “Conclusion”.

## Solar PV model

**Figure 1:**
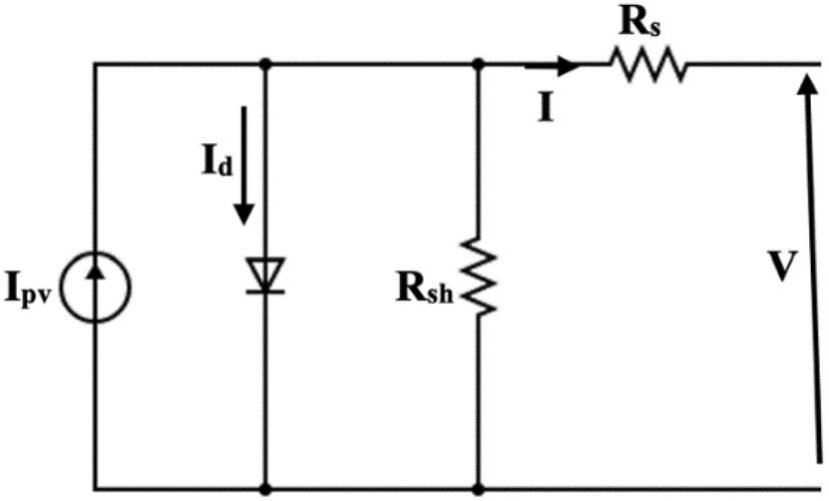
The SEM model of solar PV.

The electrical model of solar PV, also term as single exponential model (SEM), is shown in Fig. 1^1^.

The solar insolation converted in electrical energy and the non-linear characteristics of solar PV have been represented by connecting current source (*I*_*pv*_) in parallel with the diode. The losses, existing in the system, are represented by series and shunt resistance, i.e., *R*_*s*_ and *R*_*sh*_. To improve the accuracy of the model, double and triple diode models have also been addressed in several works. However, due to simplicity and accuracy similar to double diode models, SEM has been considered in most of the researches^17^. Therefore, in this study, the SEM of solar PV is used. change in irradiation or insolation conditions. The characteristic equation of the SEM is given by Eq. (1).

1 $$I = I_{pv} - I_{s} \left[ {\exp \left( {\frac{{V + IR_{s} }}{{AV_{T} }}} \right) - 1} \right] - \left( {\frac{{V + IR_{s} }}{{R_{sh} }}} \right)$$

where *V*_*T*_ is the thermal voltage (= *KT*/*q*), *K* is the Boltzmann constant, *T* is the temperature in Kelvin and *q* is the electron charge. The five unknown model parameters of the SEM are *I*_*pv*_*, I*_*s*_*, R*_*s*_*, R*_*sh*_*,* and* A*. The major of estimation of these unknown parameters is the non-linear characteristics of (1).

## Development of the objective function based on datasheet

In most of the studies the unknown parameters of a PV cell/module are estimated by minimizing an objective function. Usually, the objective function in most of the studies, minimizes the error, existing between desired and estimated characteristics, through some optimization techniques such as, Newton Raphson or Metaheuristic algorithms. The Root mean square error (RMSE), absolute relative error of current and voltage have been used as an objective-functions. However, such objective functions need experimental *I–V* data. Therefore, objective functions other than RMSE, and absolute relative error should be developed for parameter estimation. In this study the parameters given in the manufacturer’s datasheet, namely *I*_*sc*_*, V*_*oc*_*, I*_*mpp*_, and *V*_*mpp*_, have been used in developing an objective function. In the development of an objective function, most reliable parameters, namely *I*_*sc*_*, V*_*oc*_*, I*_*mpp*_, and *V*_*mpp*_, given in datasheet, have been used in the designing the objective function. Here, these parameters are determined in the controlled environmental condition by the manufacturer with the high quality of lab-setup and hence termed as ‘reliable data’. It should be noted that the proper selection and development of an objective function, decide the simplicity, speed, robustness, and accuracy of the proposed parameter estimation method. Further, the objective function in this study is developed as follows:

Firstly, the characteristic equation under the SCC, OCC, and MPP are derived and given as:

2 $$I_{sc} = I_{pv} - I_{s} \left[ {\exp \left( {\frac{{I_{sc} R_{s} }}{{N_{s} AV_{T} }}} \right) - 1} \right] - \frac{{I_{sc} R_{s} }}{{R_{sh} }}$$

3 $$I_{pv} - I_{s} \left[ {\exp \left( {\frac{{V_{oc} }}{{N_{s} AV_{T} }}} \right) - 1} \right] - \frac{{V_{oc} }}{{R_{sh} }} = 0$$

4 $$I_{mpp} = I_{pv} - I_{s} \left[ {\exp \left( {\frac{{V_{mpp} + I_{mpp} R_{s} }}{{N_{s} AV_{T} }}} \right) - 1} \right] - \frac{{V_{mpp} + I_{mpp} R_{s} }}{{R_{sh} }}$$

where *N*_*s*_ is the number of cells in a series. Secondly, the Eqs. (2)–(4) are rewritten to obtain three functions *O*_*1*_*(x), O*_*2*_*(x),* and* O*_*3*_*(x)*, respectively.

5 $$O_{1} \left( x \right) = I_{pv} - I_{s} \left[ {\exp \left( {\frac{{I_{sc} R_{s} }}{{N_{s} AV_{T} }}} \right) - 1} \right] - \frac{{I_{sc} R_{s} }}{{R_{sh} }} - I_{sc} = 0$$

6 $$O_{2} \left( x \right) = I_{pv} - I_{s} \left[ {\exp \left( {\frac{{V_{oc} }}{{N_{s} AV_{T} }}} \right) - 1} \right] - \frac{{V_{oc} }}{{R_{sh} }} = 0$$

7 $$O_{3} \left( x \right) = I_{pv} - I_{s} \left[ {\exp \left( {\frac{{V_{mpp} + I_{mpp} R_{s} }}{{N_{s} AV_{T} }}} \right) - 1} \right] - \frac{{V_{mpp} + I_{mpp} R_{s} }}{{R_{sh} }} - I_{mpp}$$

*O*_*1*_*(x), O*_*2*_*(x),* and* O*_*3*_*(x)* are the function of five unknown parameters $I_{pv} ,{ }I_{s} ,{ }R_{s} ,{ }R_{sh} ,{ }and{ }A$. Lastly, the objective function is developed by taking the non-linear least square of *O*_*1*_*(x), O*_*2*_*(x),* and* O*_*3*_*(x)* and given as:

8 $$O\left( x \right) = \left[ {O_{1}^{2} \left( x \right) + O_{2}^{2} \left( x \right) + O_{3}^{2} \left( x \right)} \right]$$

The developed datasheet-based objective function given in Eq. (8) is minimized via PSO and HS, which are briefly explained below.

### Fundamentals of PSO

The PSO algorithm is inspired from the food-searching process of the school of birds and fish. The manner in which the collective effort of birds searches the food is mimicked to search the global minima or maxima of any problem. Thus, PSO is an agent-based search optimization^18^. The important tunning parameters, in PSO, are the population size of agents, the maximum number of iterations, the learning factor, *c*_*1*_ and* c*_*2*_, arbitrary number *r*_*1*_, *r*_*2*_, and inertia weight, *w*. Initially, the population of search agents, called particles, is initialized. These agents search the search space for the minimum value. The minima among the agents are assigned as global minima*, i.e.,* ‘*G*_*best*_’. Based on this, position vector *x*_*i*_*(t)* and velocity vector *v*_*i*_*(t)* of agents are defined in every iteration. The vector *x*_*i*_*(t)* and *v*_*i*_*(t)* is updated using (9) and (10), respectively. In every iteration, the *G*_*best*_ is updated by comparing the present minima *P*_*best*_ of each iteration. Further, the objective function is tested for the *G*_*best*_ solution. The process is repeated till the optimal solution is achieved^18^.

9 $${\text{v}}_{{\text{i}}} \left( {{\text{t}} + 1} \right) = {\text{w}}\left( {\text{t}} \right){\text{v}}_{{\text{i}}} \left( {\text{t}} \right) + {\text{r}}_{1} {\text{c}}_{1} \left( {\text{t}} \right)\left[ {{\text{P}}_{{{\text{best}},{\text{i}}}} \left( {\text{t}} \right) - {\text{x}}_{{\text{i}}} \left( {\text{t}} \right)} \right] + {\text{r}}_{2} {\text{c}}_{2} \left( {\text{t}} \right)\left[ {{\text{G}}_{{{\text{best}},{\text{i}}}} \left( {\text{t}} \right) - {\text{x}}_{{\text{i}}} \left( {\text{t}} \right)} \right]$$

10 $$x_{i} \left( {t + 1} \right) = v_{i} \left( {t + 1} \right) + x_{i} \left( t \right).$$

### Fundamentals of harmony search (HS)

**Figure 2:**
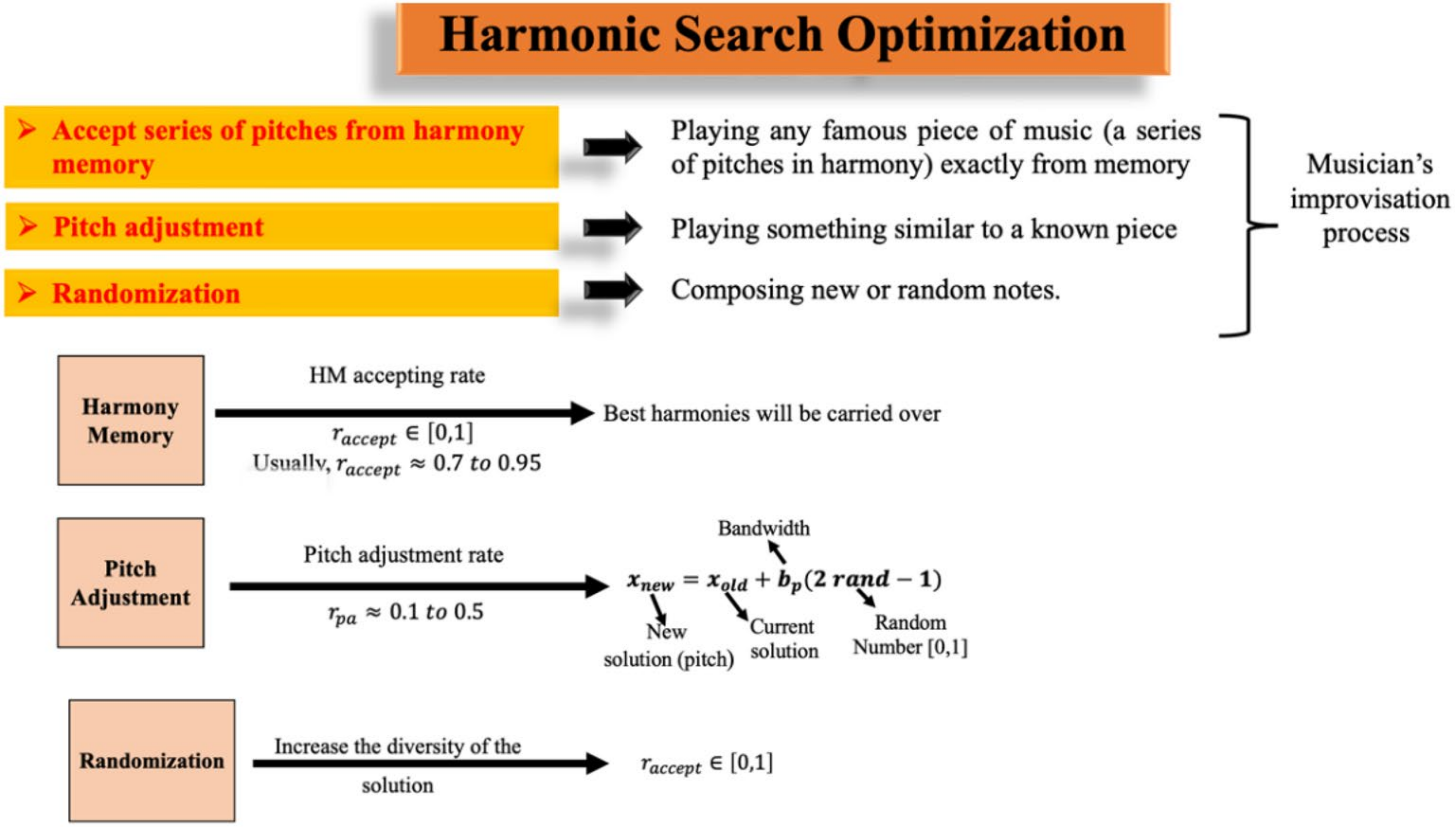
Flow diagram of HS algorithm.

The HS algorithm is inspired by the musician improvising their music^18^. The HS is a random optimization algorithm that searches for the minima by generating a harmony memory. The new solution, called pitch, is adjusted. Also, the algorithm uses randomization to avoid trapping in local minima. The flow diagram of HS algorithm is shown in Fig. 2.

## Results and discussions

The effectiveness and preciseness of the developed method are tested by considering two PV modules namely, Kyocera KD210GH-2PU (poly-crystalline), and Shell SQ85 (mono-crystalline). The details of these two modules, which are provided by the manufacture, are summarized in Table 1^19,20^.

**Table 1:** STC parameters of solar PV modules.

Parameters	Kyocera KD210GH-2PU	Shell SQ85
*V*_*oc*_* (Ω)*	33.20	22.20
*I*_*sc*_	8.58	5.45
*V*_*mpp*_	26.60	17.20
*I*_*mpp*_	7.90	4.95
*P*_*mpp*_	210.140	85.140
*K*_*v*_* (V/*^*o*^*C)*	− 1.20 × 10^–1^	− 72.5 × 10^–3^
*K*_*I*_* (A/*^*o*^*C)*	5.15 × 10^–3^	0.8 × 10^–3^
*K*_*mpp*_* (%/*^*o*^*C)*	− 0.45	− 0.43
*N*_*s*_	54	36

The search range of unknown parameters is chosen the same for PSO and HS. Table 2 indicates the selected lower and upper bound for the unknown model parameters. The range is selected according to the characteristics of mono- and poly-crystalline material.

1. The value of *I*_*pv*_ is near the value of *I*_*sc.*_
2. The value of *R*_*s*_ is very small.
3. The value of *R*_*sh*_ is comparatively greater than R_s_ in the range of 10–100 ohms.
4. The value of *A* is found to be between 1 and 2 for all the types of PV modules.

**Table 2:** Upper and lower range of unknown parameters used in PSO and HS. Significant values are in italics.

	Kyocera KD210GH-2PU	Shell SQ85
*R*_*s*_* (Ω)*	0.001, 1	0.001, 0.1
*R*_*sh*_* (Ω)*	50, 200	1, 100
*I*_*pv*_* (A)*	8, 8.7	5.4, 5.6
*I*_*o*_* (A)*	*1e*^*−5*^*, 7e*^*−8*^	*1e−5, 1e−8*
*A(*= *aV*_*t*_*N*_*s*_*)*	*1, 2*	*1, 2*

The values of tuning parameters of PSO algorithms are as follows: population size (N) = 70,000, maximum generation to be evaluated = 1000, members of particle = 5, maximum inertia weight factor ($\omega_{max}$) = 0.9, minimum inertia weight factor ($\omega_{min}$) = 0.4, acceleration constant $c_{1}$ and $c_{2}$ = 2, and for the HS algorithm, pitch limits = 0.1–0.7, pitch adjusting rate ($r_{pa}$) = 0.7, harmony memory accepting rate, ($r_{accept}$) = 0.95, maximum iteration = 1000.

### Implementation of proposed method on poly-crystalle PV module

The developed datasheet-based parameter estimation method is used to find the unknown model parameters of KD210GH-2PU. The accuracy and efficacy of the developed method are tested by estimating unknown model parameters through PSO and HS. The iteration numbers, tunning parameters, and search range are kept same in both PSO and HS in the determination of the unknown parameters. The five iteration results, achieved with developed method, using PSO and HS are shown in Tables 3 and 4, respectively. The percentage maximum power deviation index (*%MPDI*) and overall model deviation index (*OMDI*) are estimated using (11) and (12) and summarized in Table 5. Here, the suffix ‘*actual*’ and ‘*est*’ represent the data given in manufacturer data sheet and estimated value, respectively.

11 $${\text{\% }}MPDI = \frac{{MPP_{actual} - MPP_{est} }}{{MPP_{actual} }} \times 100$$

12 $$OMDI = [\left( {P_{mpp} - P_{mpp,est} } \right) + \left( {I_{mpp} - I_{mpp,est} } \right) + \left( {I_{sc} - I_{sc,est} { }} \right) + \left( {V_{oc} - V_{oc,est} } \right)$$

**Table 3:** Unknown model parameters of KD210GH-2PU obtained via the developed method using PSO.

Kyocera KD210GH-2PU, PSO Algorithm					
Parameters	Iterations				
	1st	2nd	3rd	4th	5th
*R*_*s*_* (Ω)*	0.001	0.001	0.0035	0.0062	0.001
*R*_*sh*_* (Ω)*	50	50	50	50	50
*I*_*pv*_* (A)*	8.5771	8.5791	8.5788	8.58	8.5787
*I*_*o*_* (A)*	1e−08	1e−08	1e−08	1e−08	1e−08
*V*_*th*_ = $N_{s} AV_{T}$	1.6140	1.6140	1.6140	1.6140	1.6140
*P*_*mpp, est*_	210.042	210.095	210.028	209.996	210.085
*I*_*sc,est*_	8.57	8.58	8.58	8.58	8.58
*V*_*oc,est*_	33.02	33.03	33.03	33.03	33.03
*I*_*mpp,est*_	7.89	7.90	7.89	7.89	7.90

**Table 4:** Unknown model parameters of KD210GH-2PU obtained via the developed method using HS.

Kyocera KD210GH-2PU, HS Algorithm					
Parameters	Iterations				
	1st	2nd	3rd	4th	5th
*R*_*s*_* (Ω)*	0.040	*0.0330*	*0.0149*	*0.0164*	*0.0296*
*R*_*sh*_* (Ω)*	52.049	*50.822*	*51.883*	*49.873*	*56.384*
*I*_*pv*_* (A)*	8.6839	*8.6959*	*8.6320*	*8.6859*	*8.6508*
*I*_*o*_* (A)*	*3.08e*^*−7*^	*2.87e*^*−7*^	*4.36e*^*−7*^	*5.36e*^*−7*^	*1.82e*^*−7*^
*V*_*th*_	*1.9365*	*1.9271*	*1.9768*	*1.9995*	*1.8766*
*P*_*mpp, est*_	*208.166*	*208.457*	*207.163*	*207.511*	*209.437*
*I*_*sc,est*_	*8.67*	*8.69*	*8.63*	*8.68*	*8.64*
*V*_*oc,est*_	*33.04*	*33.02*	*33.03*	*33.00*	*33.01*
*I*_*mpp,est*_	*7.82*	*7.83*	*7.79*	*7.80*	*7.87*

Significant values are in [italics].

**Table 5:** Performance Index of Kyocera KD210GH-2PU.

Perform. Index		1st	2nd	3rd	4th	5th
PSO	$\%MPDI$	0.0466	**0.0214**	0.0532	0.0685	0.0261
	*OMDI*	0.298	**0.215**	0.292	0.324	0.225
HS	$\%MPDI$	0.9393	0.8008	1.4166	1.2510	**0.3345**
	*OMDI*	2.304	2.043	3.307	3.029	**0.983**

Significant values are in [bold].

From the Tables 3, 4 and 5, following observations can be highlighted:

1. After each iteration, the developed method with PSO provides more uniform results than the HS (Tables 3, 4). For instance, *R*_*sh*_ is found 50 Ω after each iteration with PSO whereas, its values change from 50 to 56 Ω with HS. The constant value of unknown parameter after each iteration shows that the developed method with PSO has more reliable prediction of unknown parameters than the HS.
2. Further, the computational complexity and time required in estimating the unknown model parameters, will be small due to a smaller number of iterations.
3. The value of *%MPDI* is found only 0.02% with PSO where as it is 0.3% with HS. However, the values of *%MPDI* provided by both the algorithm, i.e*.*, PSO and HS are very small but the PSO outperforms the HS. The small value of *%MPDI* indicates the improved accuracy and efficacy of the developed method (Table 5).
4. The small value of *OMDI*, provided by the developed method with PSO, indicates that the solar PV characteristics, obtained through developed method or model, closely in tune with the real characteristics (Table 5).
5. The developed technique is reliable due to the small value of *OMDI*, i.e*.,* less than unity.

**Figure 3:**
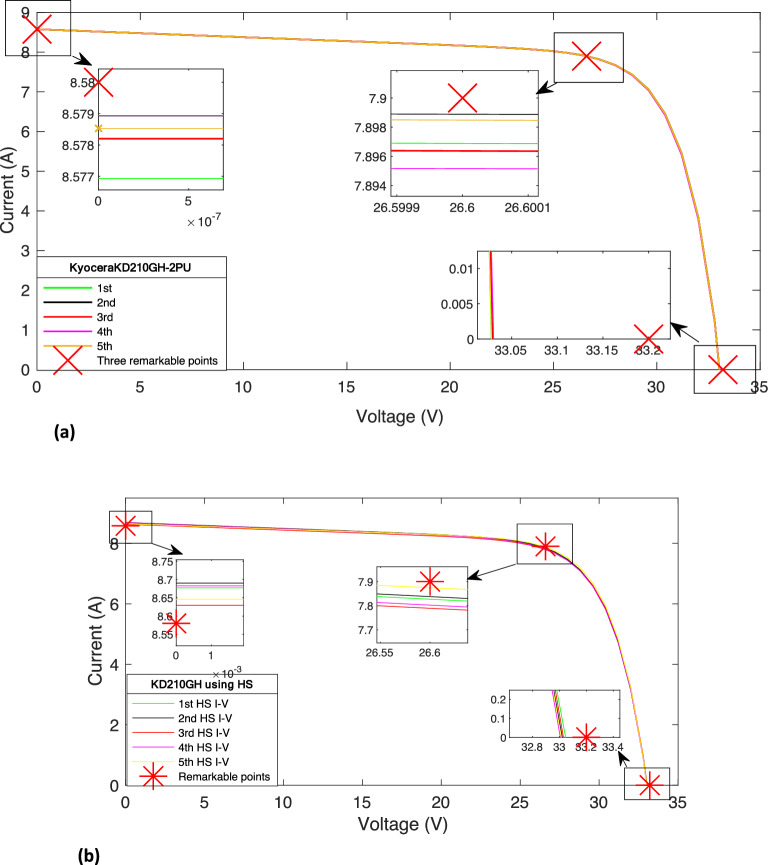
(**a**) The *I–V* characteristic curve of KD210GH using PSO. (**b**) The *I-V* curve of KD210GH using HS.

**Figure 4:**
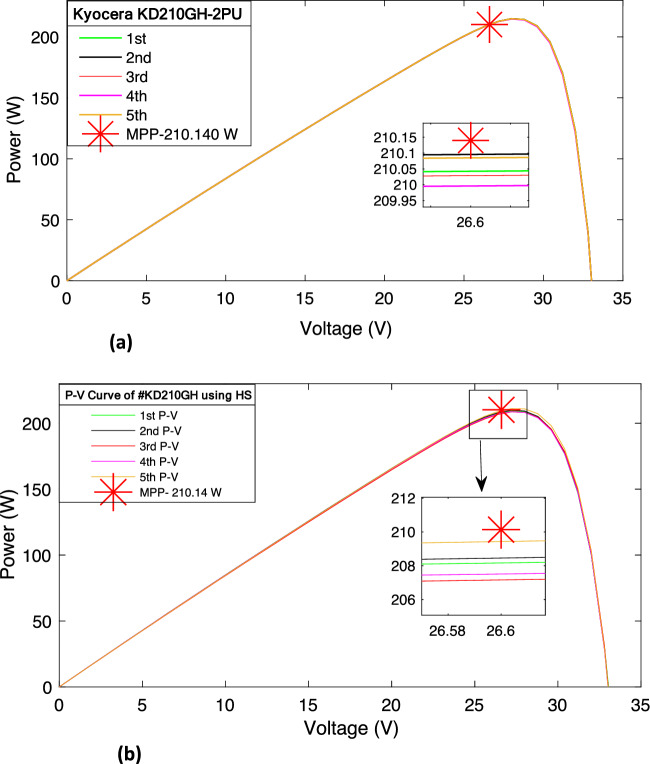
(**a**) The *P–V* characteristic curve of KD210GH using PSO. (**b**) The *P–V* curve of KD210GH using HS.

Also, using the knowledge of model parameter, as summarized in Tables 3 and 4, the *I–V* and *P–V* characteristics curves of solar PV, namely KD210GH PV module, are obtained and are shown in Figs. 3 and 4, respectively. The *I–V* curve obtained with PSO passes through three remarkable points (*V*_*oc*_ = 33.03, *I*_*oc*_ = 0), (*V*_*sc*_ = 0, *I*_*sc*_ = 8.58), and (*V*_*mpp*_ = 26.60, *I*_*mpp*_ = 7.90) whereas the same with HS passes through the points (*V*_*oc*_ = 33.01, *I*_*oc*_ = 0), (*V*_*sc*_ = 0, *I*_*sc*_ = 8.64), and (*V*_*mpp*_ = 26.60, *I*_*mpp*_ = 7.87). These remarkable points, as obtained with developed method with PSO and HS, have been considered as the ‘estimated values’ in (11) and (12) for the estimation of *%MPDI* and *OMDI.*

**Figure 5:**
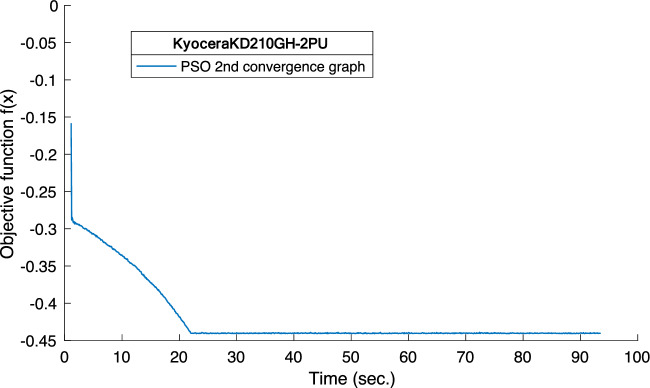
The convergence curve of PSO for KD210GH-2PU.

It is evident from the discussion, as presented through the comparative analysis between developed method with PSO and HS, it can be said that the developed method with PSO outperforms HS. Further, to check the speed of convergence of PSO, convergence curve is also obtained and it is depicted in Fig. 5. It is obvious that the developed technique achieved the steady state value in 20 s, only.

In real-time operation, the solar PV modules are subjected to a changing environmental condition^21,22^. The change in the operating temperature and irradiance affects the output *I–V* and *P–V* characteristics of a solar PV cell^21,22^. Hence it is important to know the actual *I–V* and *P–V* characteristics of a solar PV cell under changing environmental conditions for accurate control of a solar PV plant. In the majority of the studies, the value of ‘*I*_*ph*_*’ and ‘I*_*o*_*’* are considered as constant. In strictly speaking, the assumption of constant values of these parameters under varying environment condition is not true. The value of *I*_*o*_*(T)* and *I*_*ph*_*(T)* at temperature *T* are calculated using Eqs. (13)–(18)^23^.

13 $$I_{o} \left( T \right) = \frac{{I_{sc} \left( T \right)}}{{\exp \left( {\frac{{V_{oc} \left( T \right)}}{{N_{s} V_{t} a}}} \right) - 1}}$$

14 $${\text{I}}_{{{\text{ph}}}} \left( {\text{T}} \right) = \left( {{\text{I}}_{{{\text{ph}},{\text{STC}}}} + {\text{K}}_{{\text{i}}} \Delta {\text{T}}} \right){\text{G}}$$

15 $$I_{sc} \left( T \right) = I_{sc} + K_{i} \Delta T$$

16 $${\text{V}}_{{{\text{oc}}}} \left( {\text{T}} \right) = {\text{V}}_{{{\text{oc}}}} + {\text{K}}_{{\text{v}}} \Delta {\text{T}}$$

17 $$I_{mpp} \left( T \right) = I_{mpp} + K_{i} \Delta T$$

18 $$V_{mpp} \left( T \right) = V_{mpp} + K_{v} \Delta T$$

where, left side of Eqs. (13)–(18) represents the value of short circuit current, open circuit voltage, current at MPP, and voltage at MPP at temperature *T*, respectively. The values of unknown parameters at different temperature are estimated using (13)–(18) and are summarized in Table 6.

**Table 6:** Value of KD210GH-2PU at varying temperature.

*R*_*s*_* (Ω)*	*R*_*sh*_*(Ω)*	*I*_*pv*_* (A)*	*I*_*o*_* (A)*	*V*_*th*_	*I*_*sc*_*(T)*	*V*_*oc*_*(T)*
T = 75 °C and G = 1 kW/m^2^						
*0.001*	*50*	8.8366	4.2399e^−7^	*1.6140*	***8.8375***	***27.2***
T = 50 °C and G = 1 kW/m^2^						
*0.001*	*50*	8.7078	6.5125e^−8^	*1.6140*	8.7087	30.2
T = 25 °C and G = 1 kW/m^2^						
*0.001*	*50*	*8.5791*	*1e−08*	*1.6140*	8.58	33.20

Significant values are in [bold and italics].

**Figure 6:**
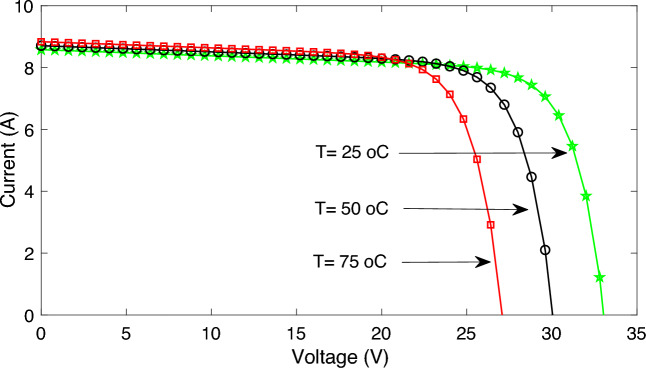
The *I–V* characteristic curve obtained by the PSO algorithm for KD210GH at different temperatures.

The *I–V* characteristics curves of KD210GH-2PU at this different temperature are shown in Fig. 6. The markers, as shown in Fig. 6, indicate the experimental data.

It is obvious from Fig. 6, the I–V characteristics curves at different temperatures, obtained with the developed method along with PSO, are exactly passing through the points which are obtained through the experimental set-up.

### Implementation of proposed method on mono-crystalline PV module

Further, to evaluate the efficacy and accuracy of the developed technique a mono-crystalline module, the Shell SQ85 has been selected for the investigation. The unknown model parameters of this module have been determined in the same way as estimated for the poly-crystalline material and summarized in Tables 7 and 8.

**Table 7:** Unknown model parameters of Shell SQ85 obtained via the developed method using PSO.

Shell SQ85, PSO Algorithm					
Unknown	Iterations				
	1st	2nd	3rd	4th	5th
*R*_*s*_***(Ω)***	0.001	0.001	0.001	0.001	0.001
*R*_*sh*_***(Ω)***	50	*50*	*50*	*50*	*50*
*I*_*pv*_ *(A)*	5.4474	*5.4503*	*5.4479*	*5.4490*	*5.4501*
*I*_*o*_ *(A)*	*7.0e*^*−7*^	*7.0e*^*−7*^	*7.0e*^*−7*^	*7.0e*^*−7*^	*7.0e*^*−7*^
*V*_*th*_	*1.3991*	*1.3991*	*1.3991*	*1.3991*	*1.3991*
*P*_*mpp*_	85.14	85.14	85.14	85.14	85.14
*n*	*700,000*	*700,000*	*700,000*	*700,000*	*700,000*
*iterations*	*1000*	*1000*	*1000*	*1000*	*1000*
*P*_*mpp,est*_	*85.01*	*85.06*	*85.02*	*85.03*	*85.05*
*I*_*sc,est*_	*5.447*	*5.45*	*5.447*	*5.448*	*5.45*
*V*_*oc,est*_	*22.026*	*22.027*	*22.027*	*22.027*	*22.027*
*I*_*mpp,est*_	*4.943*	*4.946*	*4.944*	*4.945*	*4.946*

Significant values are in [italics].

**Table 8:** Unknown model parameters of Shell SQ85 obtained via the developed method using HS.

Shell SQ85, HS Algorithm					
Unknown	Iterations				
	1st	2nd	3rd	4th	5th
*R*_*s*_ ***(Ω)***	0.0717	*0.0901*	*0.0759*	*0.0839*	*0.0825*
*R*_*sh*_ ***(Ω)***	46.0866	*46.6980*	*46.3504*	*46.6777*	*46.5331*
*I*_*pv*_ *(A)*	5.4905	*5.4991*	*5.4958*	*5.4827*	*5.4848*
*I*_*o*_ *(A)*	*6.7637e*^*−7*^	*5.2445e*^*−7*^	*9.2437e*^*−7*^	*4.8380e*^*−7*^	*3.9738e*^*−7*^
*V*_*th*_	*1.3953*	*1.3726*	*1.4233*	*1.3667*	*1.3519*
*P*_*mpp*_	85.14	85.14	85.14	85.14	85.14
*iterations*	*10,000*	*10,000*	*10,000*	*10,000*	*10,000*
*P*_*mpp,est*_	*84.34*	*84.47*	*84.18*	*84.36*	*84.58*
*I*_*sc,est*_	*5.482*	*5.488*	*5.487*	*5.473*	*5.475*
*V*_*oc,est*_	*22.036*	*22.031*	*22.038*	*22.041*	*22.07*
*I*_*mpp,est*_	*4.90*	*4.91*	*4.89*	*4.90*	*4.92*

Significant values are in [italics].

**Figure 7:**
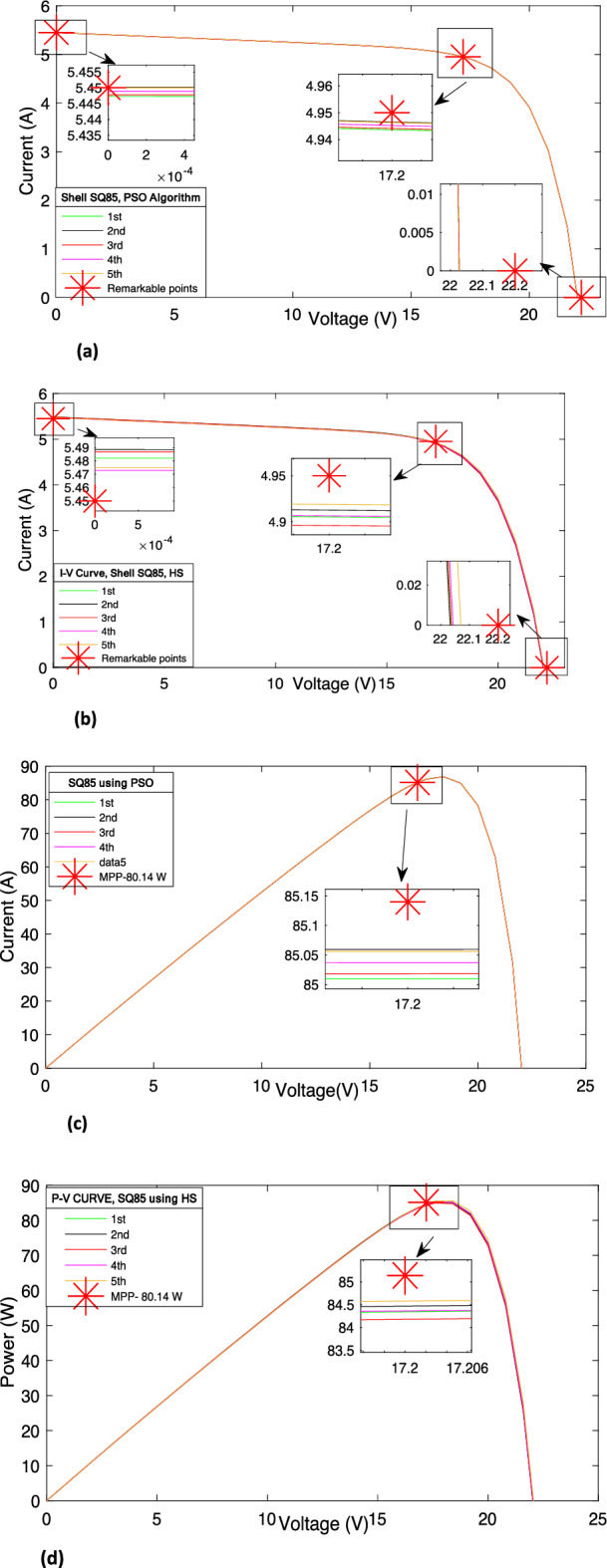
(**a**) The *I–V* characteristic curve of SQ85 using PSO. (**b**) The *I–V* characteristic curve of SQ85 using HS. (**c**) The *P–V* characteristic curve of SQ85 using PSO. (**d**) The *P–V* characteristic curve of SQ85 using HS.

Almost similar patterns are obtained for the mono-crystalline module, also. The developed method with PSO provides more uniform results than the HS after each iteration. Therefore, the developed method is found suitable for mono-crystalline module and follow all the attributes as discussed in section “Implementation of proposed method on poly-crystalle PV module” for poly crystalline module. Moreover, using the data, as summarized in Tables 7 and 8, the *I–V* and *P–V* characteristic curves are obtained for the Shell SQ85 PV module and shown in Fig. 7a–d.

Again, it is obvious from the Fig. 7a–d, the developed method with PSO outperforms the HS. For instance, *V*_*oc*_, given in datasheet (Table 1), is 22.20 and the same is estimated with developed method with PSO is 22.027. The percentage error, calculated with estimated and measured value for *V*_*oc*_, is found only 0.77%, only. Similarly, the percentage differences between estimated and measured value of other parameters, i.e*.*,* I*_*oc*_, *I*_*sc*_, *V*_*mpp*_, and *I*_*mpp*_ are found smaller with PSO than HS. Therefore, it can be said that developed method is found more suitable with PSO than the HS.

**Figure 8:**
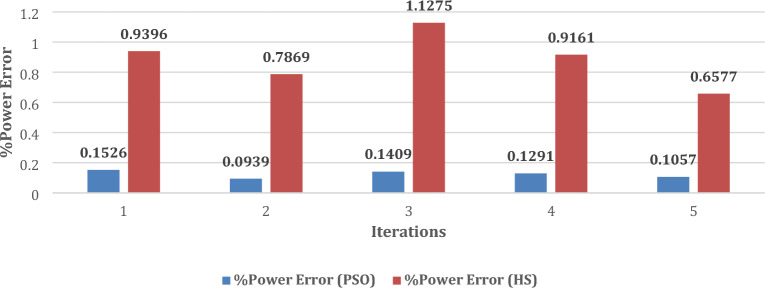
Change in *%MPDI* after each iteration.

**Figure 9:**
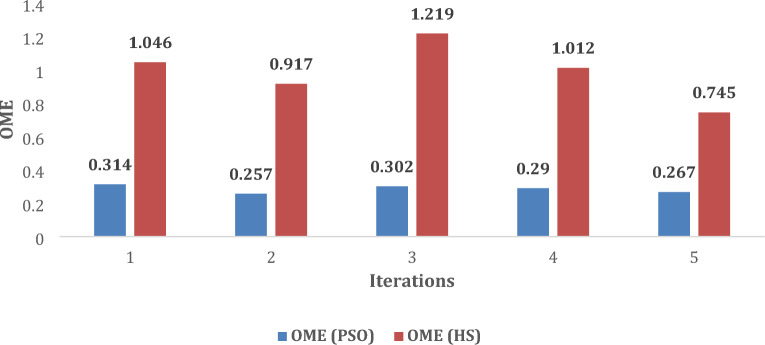
Change in *OMDI* after each iteration.

Moreover, the values of performance indices, namely *%MPDI* and *OMDI* have been estimated using the data obtained from Fig. 7a and d and Eqs. (11) and (12). The value of these performance indices, obtained with developed method using PSO and HS, are summarized in Table 9. It is obvious from the results, as summarized in Table 9, the minimum values of *%MPDI* obtained with PSO and HS, are 0.0939 and 0.6577, respectively. Further, Fig. 8 shows that almost uniform value of *%MPDI* is provided by the developed method with PSO after each iteration. The merits of uniform and small value of *%MPDI*, has already been discussed in section “Implementation of proposed method on poly-crystalle PV module”. Further, the statistical analysis of *%MPDI* with respect to number of iterations is shown in Fig. 8. It is evident that the value of *%MPDI* provided by the developed method along with PSO is almost lies on the straight line, i.e*.,* almost uniform after each iteration. Further, statistical analysis of *OMDI* has also been shown in Fig. 9 to check the efficacy of the developed technique with PSO and HS.

**Table 9:** Performance Index of Shell SQ85.

Performance Index		1st	2nd	3rd	4th	5th
PSO	*%MPDI*	0.1526	**0.0939**	0.1409	0.1291	0.1057
	*OMDI*	0.314	**0.257**	0.302	0.29	0.267
HS	*%MPDI*	0.9396	0.7869	1.1275	0.9161	**0.6577**
	*OMDI*	1.046	0.917	1.219	1.012	**0.745**

Significant values are in [bold].

From the statistical analysis, as shown in Figs. 8 and 9, the following observations can be highlighted:

1. Both *%MPDI and OMDI* are smaller in case of developed method with PSO than the HS. The small values of the *E*_*OME*_ indicates that characteristics of solar PV, i.e., Shell SQ85, as estimated with developed method, is very close to the real characteristics and has lower uncertainty. Further, the smaller value of *%MPDI* indicates good tracking capability of MPP.
2. The maximum variations in *%MPDI* is found 0.05% with PSO whereas it is 0.47% with HS. This indicates that the speed to achieve steady state value is very fast. Stated differently, the developed method takes only in few iterations for calculating unknown variables and hence fast.
3. As the *%MPDI* and *OMDI*_,_ both are found very small and hence the characteristics curve obtained with the developed method will be very near to the real characteristics.

**Figure 10:**
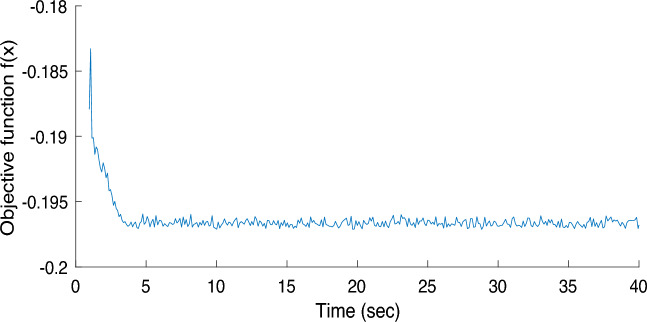
The convergence graph of SQ85 using PSO.

**Figure 11:**
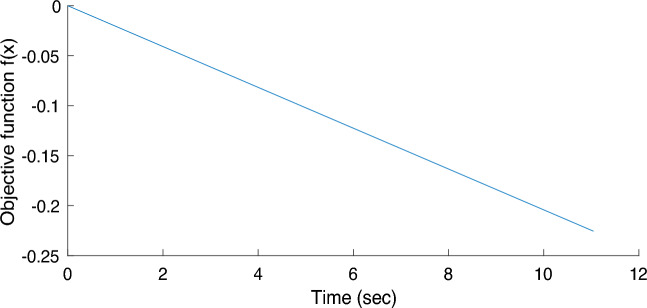
The convergence graph of SQ85 using HS.

Additionally, the convergence rate of PSO and HS is shown in Figs. 10 and 11. It is evident that the objective function, as developed in the developed method, takes only about 5 s in the optimization and to provide the values of unknown model parameters.

**Figure 12:**
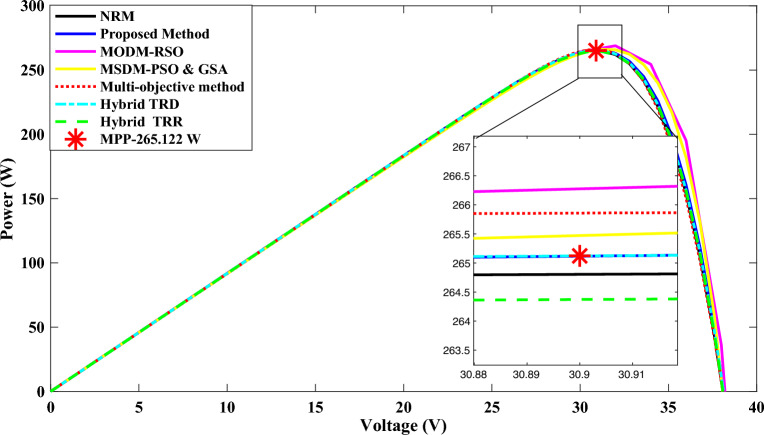
The P–V characteristic curve of H&T GmbH TS265D60 PV module.

Now the comparative analysis between the performance, as achieved from the developed technique, and existing techniques have been performed to check the efficacy and accuracy of the developed technique. For this, a PV module, namely H&T GmbH TS265D60, consisting 60 PV cells of mono-crystalline have been considered. The data given in manufacturer data sheet are 30.9 V, 8.58 A, 38.1 V, 9.19 A, and 265.122 W for *V*_*mpp*_*, I*_*mpp*_*, V*_*oc*_*, I*_*sc*_, and *P*_*mpp*_ (at STC)^1^. Using the developed and existing methods, PV curves for this module have been extracted and shown in Fig. 12. The values of estimated unknown parameters, as estimated with developed and existing techniques, are summarized in Table 10.

**Table 10:** Estimated parameters of H&T GmbH TS265D60 module via developed method, multi-objective method^22^, NRM, and hybrid TRR, TRD methods^1^.

PV module	Method	*I*_*pv*_(A)	*I*_*o*_(A)	*R*_*s*_ (Ω)	*R*_*sh*_ (Ω)	*V*_*th*_* (*= *N*_*s*_*AV*_*T*_*)*
H&T GmbH TS265D60	**Developed Method with PSO**	**9.1934**	**1.00 e**^**−07**^	**0.1703**	**876.4204**	**2.0811**
	Hybrid trust region reflective algorithm (TRR)^1^	9.1913	1.1478 e^−7^	0.1739	1216.2	0.0349
	Hybrid trust-region dogleg (TRD)^1^	9.1911	5.8865 e^−8^	0.1939	1659.4	0.0337
	Multi-objective method^22^	9.1705	9.00 e^−09^	0.2348	3844.3	1.8329
	NRM	9.1911	5.872e^−08^	0.914	1661.2	2.0195

Significant values are in [bold].

Additionally, for the comparative analysis, the modified one diode model (MODM) of solar PV cell has been used to obtain the output characteristic of PV cell. The metaheuristic algorithm namely, Rat Swarm Optimizer (RSO), and a hybrid algorithm, i.e., Particle Swarm Optimization and Gravitational Search Algorithm (PSOGSA) were used on the modified one diode model. The results obtained with the existing MODM-RSO^25^ and MODM-PSOGSA^26^ parameter estimation methods are also used to analyze the performance of the developed method.

Using the data, as summarized in Table 10 and shown in Fig. 12, the value of *E*_*ARMP*_*%* and *E*_*OME*_ are estimated and summarized in Tables 11 and 12.

**Table 11:** Absolute relative maximum power error calculated for H&T GmbH TS265D60.

Solar PV modules	Estimation methods	$MPP_{actual}$	$MPP_{est}$	*%MPDI*
H&T GmbH TS265D60	**Developed Method with PSO**	**265.122**	**265.117**	**0.004188**
	Hybrid TRR^1^	265.122	265.15	0.01056118
	Multi-objective method^24^	265.122	265.218	0.03620974
	Hybrid (Analytical)^15^	265.122	265.037	0.031796
	NRM	265.122	264.804	0.11994478
	MODM-RSO^25^	265.122	266.275	0.4348
	MODM-PSOGSA^26^	265.122	265.475	0.1331

Significant values are in [bold].

**Table 12:** Overall model error calculated for H&T GmbH.

PV modules	Extraction methods	$P_{mpp,est}$	$I_{mpp,est}$	$I_{sc,est}$	$V_{oc,est}$	*OMDI*
H&T GmbH, TS265D60	**Developed Method with PSO**	**265.117**	**8.58**	**9.19**	**38.1**	**0.005**
	Hybrid TRR method^1^	265.15	8.55	9.19	38.09	0.068
	Multi-objective method^24^	265.218	8.58	9.17	38.01	0.21
	NRM	264.804	8.57	9.19	38.09	0.348
	MODM-RSO^25^	266.275	8.62	9.20	38.21	1.313
	MODM-PSOGSA^26^	265.475	8.59	9.18	38.07	0.513

Significant values are in [bold].

It is evident from Tables 11 and 12; the values of *%MPDI* and *OMDI* are smaller with the developed method than the other existing hybrid, multi-objective, numerical method-based approaches and modified one diode method. The MODM-RSO has the highest deviation from real characteristics because of the high values of performances indices. Through the comparative analysis, as summarized in Tables 11 and 12, the following major outcomes of the developed method can be highlighted:

1. The developed technique has the smallest value of *%MPDI*. Basically, the performance index, namely *E*_*ARMP*_*%* is an indicator to show the closeness of estimated and actual MPP. The *%MPDI* of developed technique is very close to zero, i.e., 0.0041%, only. This indicates the developed technique is more accurate in estimating MPP than the other existing techniques compared here for the analysis. Further, the MPP predicted by the developed method will be very closely align to the real value.
2. Also, if reliability is considered than the parameter estimated by the developed technique is considered as the most reliable due to very small value of *E*_*ARMP*_*%*. Further, the developed technique is based on datasheet parameters, which have already been evaluated in the control environment and good quality experimental set-up, hence this enhances the reliability of the developed method. Additionally, the small value *E*_*OME*_ represents the accuracy and efficacy of the suggested model.

Therefore, before proceeding to the design part, the developed method can be considered as fast, reliable, and efficient method for the estimation of the unknown model parameters.

## Conclusion

An NLS objective function, based on manufacturer datasheet, has been developed in the presented work. Furthermore, using PSO and HS the developed objective function has been optimized to get accurate value of the unknown model parameters of solar PV. The efficacy, accuracy, and speed of the developed method has been tested by applying it on mono-and poly crystalline solar PV. It is shown that for both types of the solar PV, developed method with the PSO outperforms the HS in terms E_ARMP_% and E_OME_. It is found that the values of *E*_*ARMP*_*%* of the developed method with PSO are 0.0939 and 0.0214 for mono- and poly crystalline material based solar PV, respectively. Further, the time of estimation of developed method with PSO has also been analyzed and it is shown that the PSO provides almost constant values of unknown parameters after each and every iteration and converges very fast, i.e., in 5 s, only. Also, the finding of the developed method has been compared with the other established methods, such as multi-objective function, hybrid with TRR, hybrid with analytical, NRM, MODM-RSO and MODM-PSOGSA based approaches. It is shown that magnitude of *E*_*ARMP*_*%* is only 0.004% whereas it is maximum with MODM-RSO (= 0.4348%) and MODM-PSOGSA (= 0.1331). Similarly, *E*_*OME*_ is found to be smallest for the proposed method whereas it is maximum for the MODM-RSO and MODM-PSOGSA. Therefore, the small values of these performance indices, i.e., *E*_*ARMP*_*%* and *E*_*OME*_, with the proposed method indicate the small deviation of maximum power from the real i.e., high accuracy.

## Data Availability

The datasets used and/or analyzed during the current study available from the corresponding author on reasonable request.
